# Host acid signal controls *Salmonella* flagella biogenesis through CadC-YdiV axis

**DOI:** 10.1080/19490976.2022.2146979

**Published:** 2022-12-01

**Authors:** Weiwei Wang, Yingying Yue, Min Zhang, Nannan Song, Haihong Jia, Yuanji Dai, Fengyu Zhang, Cuiling Li, Bingqing Li

**Affiliations:** a Department of Clinical Laboratory, Shandong Provincial Hospital Affiliated to Shandong First Medical University, Jinan, China; b Department of Pathogen Biology, School of Clinical and Basic Medical Sciences, Shandong First Medical University & Shandong Academy of Medical Sciences, Jinan, China; c State Key Laboratory of Microbial Technology, School of Life Sciences, Shandong University, Qingdao, China; d Shandong First Medical University, Key Lab for Biotech-Drugs of National Health Commission, Jinan, China; e KeyLaboratory for Rare & Uncommon Diseases of Shandong Province, Jinan, China

**Keywords:** *Salmonella typhimurium*, acid signal, YdiV, CadC, FlhDC, flagellar biosynthesis, immune escape

## Abstract

Upon entering host cells, *Salmonella* quickly turns off flagella biogenesis to avoid recognition by the host immune system. However, it is not clear which host signal(s) *Salmonella* senses to initiate flagellum control. Here, we demonstrate that the acid signal can suppress flagella synthesis and motility of *Salmonella*, and this occurs after the transcription of master flagellar gene *flhDC* and depends on the anti-FlhDC factor YdiV. YdiV expression is activated after acid treatment. A global screen with *ydiV* promoter DNA and total protein from acid-treated *Salmonella* revealed a novel regulator of YdiV, the acid-related transcription factor CadC. Further studies showed that CadC_C_, the DNA binding domain of CadC, directly binds to a 33 nt region of the *ydiV* promoter with a 0.2 μM K_D_ affinity. Furthermore, CadC could separate H-NS-*ydiV* promoter DNA complex to form CadC-DNA complex at a low concentration. Structural simulation and mutagenesis assays revealed that H43 and W106 of CadC are essential for *ydiV* promoter binding. No acid-induced flagellum control phenotype was observed in *cadC* mutant or *ydiV* mutant strains, suggesting that flagellum control during acid adaption is dependent on CadC and YdiV. The intracellular survival ability of *cadC* mutant strain decreased significantly compared with WT strain while the flagellin expression could not be effectively controlled in the *cadC* mutant strain when surviving within host cells. Together, our results demonstrated that acid stress acts as an important host signal to trigger *Salmonella* flagellum control through the CadC-YdiV-FlhDC axis, allowing *Salmonella* to sense a hostile environment and regulate flagellar synthesis during infection.

## Introduction

*Salmonella* is an important facultative pathogen of many animals and humans, and *Salmonella* infection triggers acute gastroenteritis and typhoid fever in hosts.^1–3^ These diseases are responsible for more than 300,000 deaths annually, presenting a significant threat to global public health, especially in developing countries.^4–6^

Once ingested, flagella allows *Salmonella* to cross the intestinal barrier and adhere to the intestinal epithelial cells.^7–9^ The bacterial flagellum is a complex macromolecular machine whose construction requires proteins encoded by three classes of genes.^10,11^ Class I comprises two genes in a single operon, *flhDC*, the“master switch” of all other flagellar genes. The FlhD_4_C_2_ complex acts as the transcription factor for class II genes.^12,13^ Products of class II flagellar genes form the flagellar basal structure and the hook-basal body complex, and class III flagellar protein are related to filament formation, flagellar rotation, and chemotaxis.^11,14–16^ After *Salmonella* enters host cells, the flagellum becomes a prime target antigen for the host immune system. Previous studies showed that the expression of flagellar antigen FliC decreased to 1/10 the original level upon *Salmonella* entry into host cells.^17,18^ Previously, three regulators were identified to be responsible for this process, YdiV, STM1697, and YdiU. YdiV, a post-transcriptional regulator, disrupts FlhDC complex structure by binding to FlhD, thereby preventing it binding to class II promoters and targeting it for proteolysis.^19–21^ STM1697, another post-transcriptional anti-FlhDC factor, represses flagellar synthesis by preventing FlhDC from recruiting RNA polymerase to the promoter.^22^ The function of FlhDC is also regulated by post-translational modification by YdiU, a modifying enzyme that catalyzes the UMPylation of the FlhC subunit to prevent FlhDC binding to flagellar genes, thus switching off flagellar biogenesis.^23^ Although these regulators control flagella biogenesis, it is not clear which host signals *Salmonella* perceives to turn on this flagella control process.

During infection, most *Salmonella* invades into host macrophages. These macrophages act as a “Trojan horse” and carry *Salmonella* into the systemic tissue, provoking a systemic infection.^24–26^ Within macrophages, *Salmonella* survives and replicates within the *Salmonella*-containing vacuole (SCV), in which *Salmonella* faces a hostile environment, with acidic pH of 4.5 to 5.5, low concentrations of magnesium and calcium, and the presence of antimicrobial peptides, oxygen and nitrogen-free radicals.^27–30^ *Salmonella* responds to different environmental signals and modulates related genes via a variety of mechanisms, such as two-component regulatory systems (TCS). PhoP-PhoQ is one of the best-characterized TCS. In *Salmonella*, PhoQ can sense environmental signals such as free radicals, low Mg^2+^ ions, and low pH, and activate PhoP to promote the transcription of some gene clusters with functions related to invasion, intra-macrophage survival, magnesium transport, and others.^31–33^ Another TCS, EnvZ-OmpR, regulates multiple cellular function including virulence, biofilm formation, and acid tolerance in response to both acid and osmotic stress.^34–36^ *Salmonella* can regulate transcription with DNA binding proteins that act as transcription repressors or activators for target genes. H-NS is a nucleoid-associated protein and acts as a master global repressor, affecting the expression of at least 250 genes in *Salmonella* by binding to the promoters of target genes.^37–39^ The inhibitory effect of H-NS can be countered by the effects of other DNA-binding proteins that can either disrupt H-NS-DNA complexes or replace H-NS on the DNA due to higher binding affinity.^40,41^

The Cad system is an acid-inducible degradative amino acid decarboxylase system, which helps bacteria resist environmental acid stress. The Cad system consists of *cadC* and *cadBA* operons.^42–44^ CadC is a membrane-spanning transcriptional activator of the *cadBA* operon and is normally in an inactive state. Under external acidification and exogenous lysine, CadC is rapidly activated by proteolytic cleavage. The N-terminus of CadC binds to the *cadBA* promoter and activates the transcription of the *cadBA* operon.^43,45^ As a result, lysine is decarboxylated to cadaverine by CadA and cadaverine is transported out of the cell by CadB, facilitating bacterial acid tolerance.^46,47^ CadC can also regulate other proteins involved in glycolysis, energy production, and stress tolerance in *Salmonella*.^45^

In this study, we discovered that acid stress can negatively regulate the flagellar synthesis of *Salmonella*. Acid-mediated flagellar control occurs after the transcription and expression of FlhDC. The expression levels of secondary and tertiary flagellar genes were significantly decreased during acid treatment while the transcription of *flhDC* was increased during acid treatment. Acid signal increased the expression of YdiV but not STM1697 and YdiU. YdiV mutant *Salmonella* loses the phenotype of acid-mediated flagellar control. Using an *in vitro* DNA pull-down assay of *ydiV* promoter, we identified the acid-initiated transcription factor CadC as a transcription regulator of YdiV. *In vitro* data showed that the DNA binding domain of CadC directly bound to the *ydiV* promoter with a 0.2 μM K_D_ affinity. Further structural simulation and mutagenesis assays identified His43 and W106 as key amino acids for *ydiV* promoter binding. The *cadC* mutant *Salmonella* loses the acid-induced YdiV activation and flagellum control phenotypes. In summary, our results revealed that a novel pathway, CadC-YdiV-FlhDC, turns off flagella biogenesis response to host acid signal by which *Salmonella* quickly shuts down flagella synthesis to achieve immune escape within host cells.

## Results

### Salmonella switches off flagellum biogenesis during acid adaptation.

Previous studies suggested that *Salmonella* rapidly turns off flagellum synthesis after entering host cells.^22,23^ When entering macrophages, *Salmonella*-containing phagosomes acidify soon after formation. As a result, *Salmonella* survives in an acidic environment within host cells. We speculate that acid signal may allow *Salmonella* to perceive the host environment for flagellum control. To investigate this, we began to study motility and flagellum synthesis under different pH environmental conditions.

**Figure 1.**
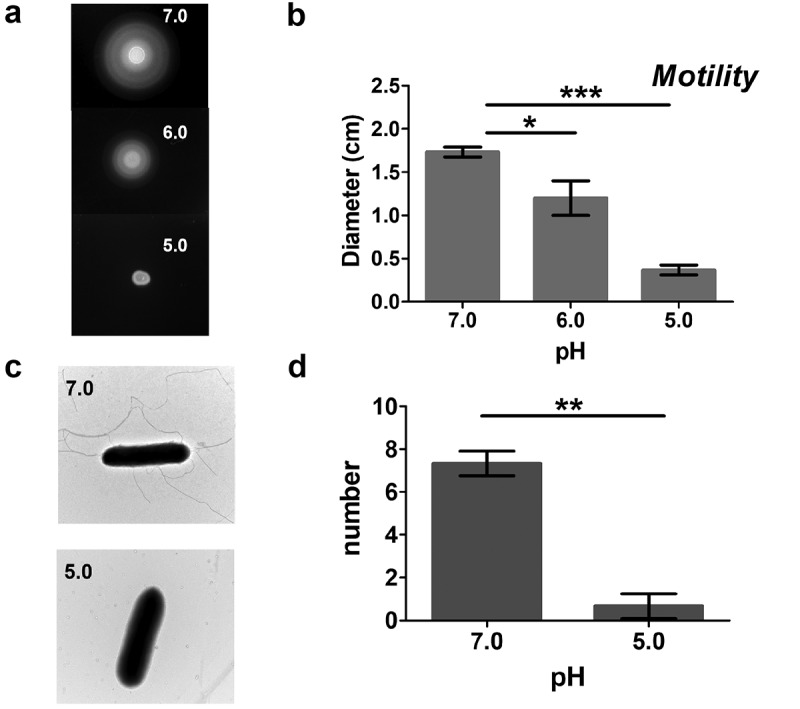
Acid stress inhibits the flagellar synthesis pathway of *Salmonella.*

The motility behavior of *Salmonella typhimurium* ATCC 14028s was first assayed using semisolid agar (0.25%) plates with different pH. The results showed that *Salmonella* exhibits normal motility at pH 7.0, however, at pH lower than 7.0, moving ability decreases, with total loss of moving ability at pH 5.0 (Figures 1a and 1b). To determine the basis of motility loss at pH 5.0, we determined the growth of flagellar filaments of *Salmonella* cultured in pH 5.0 or pH 7.0 using negative-staining electron microscopy (Figures 1c and 1d). Fewer flagella were observed in *Salmonella* cultured in pH 5.0 than those cultures in neutral growth conditions. Overall, our data demonstrate that flagellum synthesis of *Salmonella* could be significantly inhibited by acid signal.

(A and B) The motility of the wild-type strain was measured using 0.25% semisolid agar plates at pH 5, 6, and 7, respectively. (C and D) The numbers of flagella of the wild-type strain were observed using negative-staining electron microscopy (EM). Data are shown as the means and SEM, n=3. ***, P < 0.001; **, P < 0.01; *, P < 0.05; n.s., P > 0.05.

### Acid-mediated flagellum control occurs after flhDC transcription.

**Figure 2.**
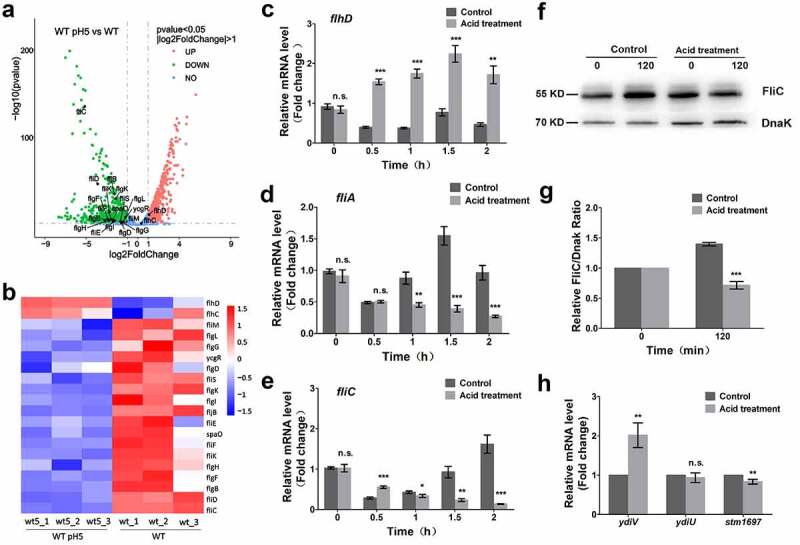
Acid stress inhibits the expression of flagellum after *flhDC* transcription.

To further probe the effect of acidity on the expression of flagellar genes in *Salmonella*, RNA-sequencing analysis was performed in *Salmonella* cultured with or without acid treatment (Figure 2 and Fig.S1). The results showed that the mRNA level was significantly increased for class I flagellar genes *flhD* and *flhC* after acid treatment (Figures 2a and 2b). However, there was severely reduced mRNA level of 18 class II and class III flagellar genes after acid treatment (Figures 2a and 2b). To verify the RNA-sequencing results, the transcription level of three flagellar genes (*flhD, fliA*, and *fliC*) of *Salmonella* before and after acid treatment was further detected by RT-PCR. The *gapdh* gene was used as an internal control and its expression was not affected by acid stress (Fig. S2). Consistent with the RNA-sequencing results, the transcription level of *flhD* was upregulated after acid treatment (Figure 2c), and the transcription level of *fliA* and *fliC* was markedly decreased under acid stress compared to under non-stress conditions (Figures 2d and 2e). Finally, the expression of FliC, the major flagellum antigen, was determined by immunoblot analysis of the treated and untreated samples. Consistent with the RT-PCR results, the protein level of FliC was significantly reduced after acid treatment (figures 2f and 2g). The above data collectively demonstrate that acid-related flagellum control occurs after class I flagellar gene *flhDC* transcription. Previous studies showed that three proteins, YdiV, YdiU, and STM1697, negatively regulate flagellum biogenesis by inhibiting the ability of FlhDC complex to regulate transcription.^20,22^ Data analysis of the above RNA-sequencing results demonstrated that the expression of *ydiV* is largely up-regulated, while expression of *ydiU* and *stm1697* was almost unchanged or even down-regulated (Figure 2h). Therefore, YdiV was chosen as the focus of further study.

(A, B and H) Differentially expressed genes of wild-type strain under normal or acid stress conditions. (A) Volcano plots were generated to visualize the transcriptomic results. Red and green points indicate upregulated and downregulated genes, respectively. Genes related to flagellar synthesis are labeled. (B) Heatmap showing relative transcript abundances of flagellar synthesis-related genes in the wild-type strain. (C to E) The expression levels of *flhD* (C), *fliA* (D), and *fliC* (E) in the wild-type strain under normal and acid stress conditions were measured by qRT-PCR. (F) The protein levels of FliC and DnaK after acid treatment were determined by Western blotting. DnaK was used as a loading control. (G) The relative FliC levels compared with DnaK were quantified by grayscale for three independent experiments. The gray values were obtained using ImageJ. (H) The relative transcription levels of flagellar regulators genes (*ydiV*, *ydiU* and *stm1697*) and *cadC* gene. Data are shown as the means and SEM, n=3. ***, P < 0.001; **, P < 0.01; *, P < 0.05; n.s., P > 0.05.

### Flagellum control during acid adaptation is dependent on YdiV.

**Figure 3.**
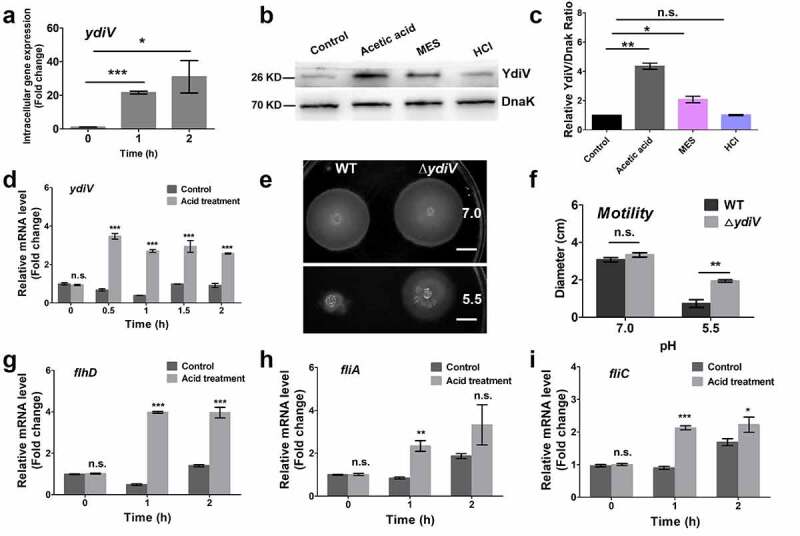
YdiV is required for flagellar control of *Salmonella* under acid stress.

The above data clearly demonstrated that the acid signal can inhibit flagellar expression in *Salmonella* and this regulation occurs after *flhDC* transcription. YdiV may be involved in the regulation of acid-flagellar inhibition in *Salmonella*. The increased expression of *ydiV* was dramatic, with increases of 21.6- and 31.0-fold 1 and 2 h after wild-type *Salmonella* entry into host cells, respectively (Figure 3a).

(A) *Salmonella* upregulates YdiV expression during invasion. The intracellular mRNA levels of ydiV before and after Salmonella invasion were detected in the wild-type strain by qRT-PCR. (B to D) The transcription and protein levels of YdiV in *Salmonella* cultivated under acid condition were detected by qRT-PCR and western blot. (B) The protein levels of YdiV in wild-type strain after normal or acid (acetic acid; 2-mercaptoethanesulfonic acid, MES; Hydrochloric acid, HCl) treatment were determined by Western blotting. DnaK was used as a loading control. (C) The relative YdiV levels compared with the levels of DnaK were quantified by grayscale in three independent experiments. The gray values were obtained using ImageJ. (D) The transcription levels of YdiV in the wild-type strain under normal and acid stress conditions were detected by qRT-PCR. (E and F) The motility of the wild-type and ∆*ydiV* strains were measured using 0.25% semisolid agar plates at pH 5.5 and 7. Scale bars shows 1 cm. (G to I) The expression levels of *flhD* (G), *fliA* (H), and *fliC* (I) in the ∆*ydiV* strain under normal and acid stress conditions were measured by qRT-PCR. Data are shown as the means and SEM, n=3. ***, P < 0.001; **, P < 0.01; *, P < 0.05; n.s., P > 0.05.

To further determine whether acid can induce the expression of *ydiV*, the protein level of YdiV was detected after acid treatment (Figures 3b and 3c). Considering that the acid stress faced by *Salmonella* in the process of invading the host includes organic and inorganic acids, we used these two different types of acids in the experiment. As expected, YdiV was barely expressed when *Salmonella* was cultured in LB medium or under inorganic acid stress (HCl), whereas expression increased to a high level under organic acid stress (acetic acid and MES). The special dissociation constant and cell membrane permeability of organic acids are better than inorganic acids. Therefore, acetic acid was selected as the acid pressure in subsequent experiment. The mRNA level of *ydiV* was detected by RT-PCR at different time points upon acid treatment, and expression increased 5.1-, 6.9-, 3.0-, and 2.8- fold, respectively, when detected 0.5, 1, 1.5, and 2 h upon acid treatment (Figure 3d). To determine whether YdiV is related to flagellum control under acidic conditions, we next assessed the expression of flagellar genes (*flhD, fliA, fliC*) in ∆*ydiV* strain during acid adaptation. The expression levels of *flhD, fliA* and *fliC* were all remarkably up-regulated after acid treatment (Figure 3g-i), indicating that ∆*ydiV* lost the acid-mediated flagellum control phenotype. Furthermore, we monitored the motility behavior of WT and ∆*ydiV* strains at different pH. There was no obvious difference in motility behavior of WT and ∆*ydiV* strains at pH7.0. However, the ∆*ydiV* did not exhibit the severe motility defect that was observed in WT strain under acidic pH condition (Figures 3e and 3f). Together, these results suggest that YdiV exerts an important role in flagellum control upon encountering acid stress.

### The acid-related transcription regulator CadC binds to ydiV promoter.

**Figure 4.**
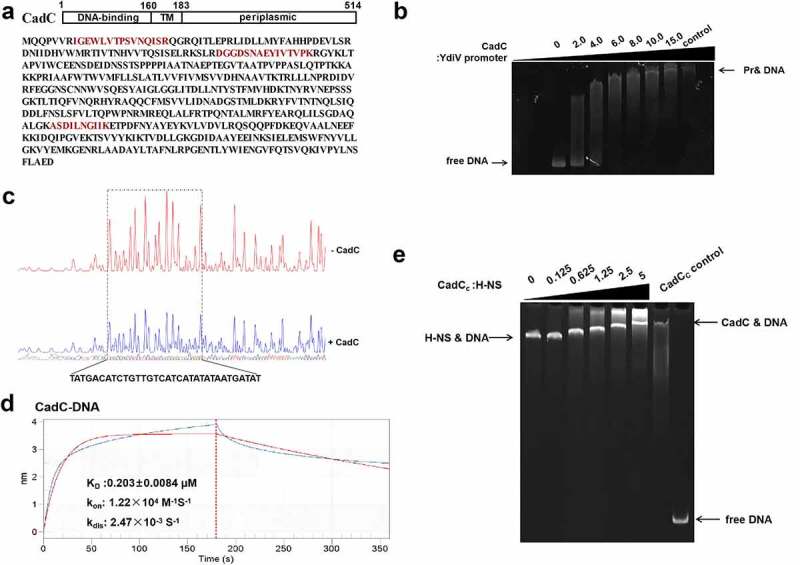
CadC binds to the promoter region of *ydiV.*

Previous studies have shown that *Salmonella* does not express YdiV in nutrient-rich medium, but nutrition deficiency or osmotic pressure signal will initiate the expression of YdiV.^20,21^ Our data revealed that acid signal up-regulated the expression of YdiV by an unknown mechanism. By analysis of genome location, we found a long non-coding region (313 bp) upstream of the *ydiV* gene (Fig. S3A), so we speculated that some transcription factors might play a regulatory role in acid-related YdiV expression. To identify the putative transcription regulator, we performed a DNA pull-down assay with the 313 bp promoter DNA region of *ydiV* and total cellular protein of *Salmonella* cultured under acidic condition (Fig. S3B). Mass spectrometry identified several transcription factors, including the acid-related regulator CadC and the global transcription repressor H-NS (Figure 4a and Fig. S4A). Previously, H-NS was found to be a positive regulator of *Salmonella* motility by silencing *ydiV* expression. However, CadC is an acid-activated transcription factor and was not previously found to be related to *ydiV*. We purified the DNA-binding domains of CadC (CadCc, CadC carboxy terminus, 1–160 amino acid) and H-NS, and confirmed their interaction with the *ydiV* promoter via electrophoretic mobility shift assay (EMSA) (Fig. S5). The EMSA results showed that both CadCc and H-NS bound to the DNA fragment, with DNA shift detected at molar ratio of CadCc: DNA above 2 and a molar ration of H-NS above 4 (Figure 4b and Fig. S4B), indicating CadCc and H-NS directly bind to the *ydiV* promoter. To more precisely determine the specific locations on the *ydiV* promoter that interact with CadC or H-NS, *in vitro* DNase I footprinting assays were performed in the presence and absence of CadCc or H-NS. The results showed that a 33 nt region of the *ydiV* promoter was protected by CadCc-binding and two regions (51 nt and 34 nt) were protected by H-NS (Figure 4c and Fig. S4C). There was a 19 nt overlap (TCATCATATATAATGATAT) region protected in the presence of both CadCc and H-NS. Furthermore, the interaction dynamics were determined with the 19 nt overlap DNA and the two proteins using the ForteBio Octet detection system. The results revealed that both CadCc and H-NS bound to this DNA with similar K_D_ values (0.203 ± 0.0084 μmoL for CadCc, 0.533 ± 0.0127 μmoL for H-NS) (Figure 4d and Fig. S4D). However, dissociation equilibrium of H-NS to target DNA was achieved much more quickly than for CadCc, with K_dis_ of 0.0145/s for H-NS and 0.00247/s for CadCc (Figure 4d and Fig. S4D), indicating the binding of CadC to target DNA is more stable than binding of H-NS. The result of EMSA competition assay further proves that CadC can dissociate the H-NS-*ydiV* promoter DNA comlex (Figure 4e). When the concentration of CadC_c_ is 18.75 µM, it begins to bind to *ydiV* promoter competitively with H-NS, and when the concentration of CadC_c_ is above 150 µM, it competes completely with H-NS to form CadC_c_-DNA complex. This data further supports our conclusion that H-NS inhibits *ydiV* transcription, while CadC removes this repression through competitive binding with *ydiV* promoter.

(A) Domain profile and sequence of CadC. Red sequence indicates the fragment identified by MS. (B) EMSA result for CadCc and *ydiV* promoter DNA; 5 µM *ydiV* promoter DNA was pre-incubated with different ratios of CadCc for 10 min. Mixtures were analyzed by native 5% polyacrylamide gel at 4 °C and then stained with GelRed. The ratio of protein to DNA ranged from 0 to 18. A sample containing only CadCc was used as a control. (C) DNase I footprinting assays of the *ydiV* promoter with CadCc. The promoter fragment of *ydiV* was PCR amplified and labeled with FAM, incubated with increasing amounts of purified CadCc, and then subjected to DNase I footprinting assay. DNase I digestion reactions were analyzed by ABI 3500XL DNA analyzer. Protected regions are boxed and marked with positions. The upper picture shows the control reactions (no added protein). Protected regions are shown in dotted squares. (D) Biolayer interferometry (BLI) analysis of the binding capacity of CadCc to overlapping DNA. Assessment of BLI response signal (nm) for CadCc protein binding to DNA. (E) CadC and H-NS compete for binding to the promoter region of *ydiV*. Competitive EMSA using the 313-bp *ydiV* promoter and the proteins H-NS (constant concentration) and CadCc (variable concentration). The sample containing CadCc+DNA and H-NS+DNA were added as control.

### H43 and W106 of CadC are essential for ydiV promoter-binding.

**Figure 5.**
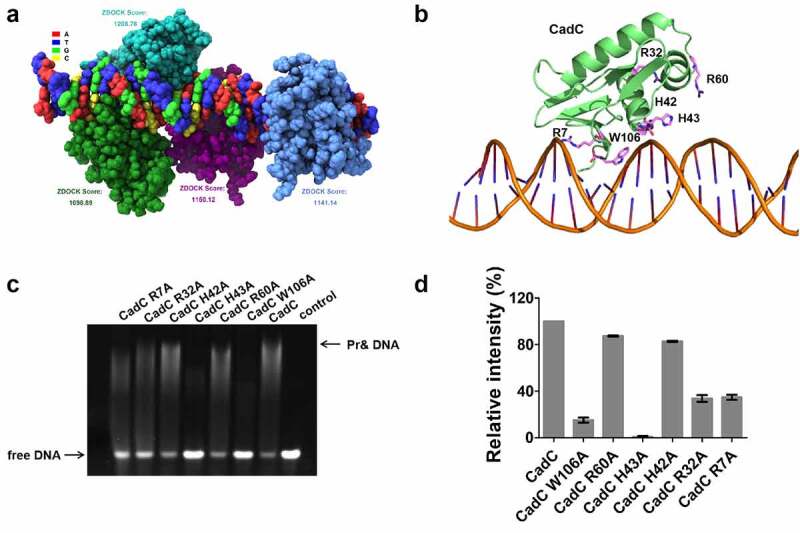
Interaction models between CadCc and target DNA.

To further characterize the interaction between CadC and target DNA, a molecular docking analysis between CadCc and target DNA was performed. To do this, a structural model of *Salmonella* CadC was built using SWISS Model based on the structure of *Escherichia coli* CadC (PDB code 5JU7), which has 76.19% amino acid sequence identity to *Salmonella* CadC. Then, the structural model of *Salmonella* CadCc-DNA complex was generated using the rigid-body docking program Zdock. The complex model of CadCc and DNA showed a good match in both shape and energy, and the highest score model contains four CadCc molecules on one target DNA molecule (Figure 5a). Further interaction surface analysis demonstrated that residues R7, R32, H42, H43, R60 and W106 of CadC could potentially interact with target DNA (Figure 5b). To determine the role of these residues in target DNA binding, six CadCc mutants (R7A, R32A, H42A, H43A, R60A, and W106A) were engineered into an expression vector and purified from *E. coli* BL21(DE3). Then, the DNA binding ability of the purified mutant proteins was investigated using EMSA (Figures 5c and 5d). Compared with native CadCc, the R7A and R32A mutants exhibited largely reduced DNA-binding ability to the *ydiV* promoter, while the H43A and W106A mutants lost almost all their DNA-binding ability to the *ydiV* promoter, indicating that H43 and W106 are the key amino acids for the CadC-*ydiV* promoter interaction. Sequence alignment of CadC homologues showed that H43 and W106 are highly conserved, indicating that the mechanism of CadC activation of *ydiV* expression under acidic conditions may be widespread in bacteria (Fig. S6). Interestingly, the *ydiV* promoter sequences of *Salmonella* and *E. coli* are completely different, and the *ydiV* promoter of *E. coli* does not have the same DNA sequence that interacts with CadC in the *Salmonella ydiV* promoter (Fig. S7). Consistent with the results of sequence alignment, EMSA results showed that both CadC_C_ and H-NS could not bind to *E. coli ydiV* promoter (Fig. S8).

(A) The model of the CadCc(DNA)_4_ complex. The four monomers of CadCc protein are colored in green, light blue, purple, and blue. The DNA molecule bound to the four CadCc monomers is constructed by bases A, T, G, and C, which are colored red, dark blue, emerald green, and yellow, respectively. (B) Detailed interactions between CadCc and DNA of the CadCc-DNA complex. The residues of the DNA binding environment (R7, R32, H42, H43, R60 and W106) are shown as sticks. (C) Binding ability of CadCc mutants to the promoter DNA, as detected by EMSA. The concentration for all CadCc proteins was 25 µM, with DNA at a concentration of 5 μM. Control: reaction system without CadCc in any form. (D) The corresponding band densitometry quantified from the results of EMSA.

### CadC is crucial for acid-mediated YdiV activation and flagellum control.

**Figure 6.**
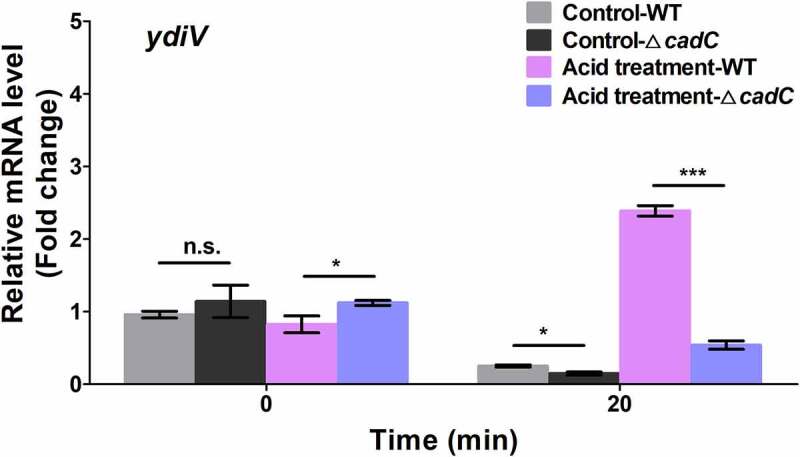
CadC exerts a positive effect on YdiV expression under acid stress.

**Figure 7.**
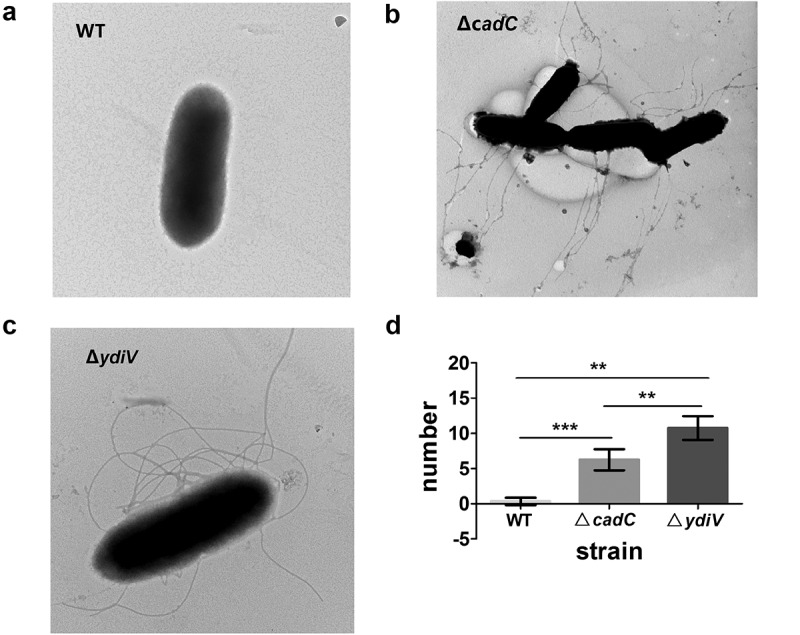
The flagellar biogenesis phenotypes of ∆*cadC* and ∆*ydiV* strains under acid stress condition.

Since our data clearly demonstrated CadCc directly binds to the *ydiV* promoter *in vitro*, we speculated that CadC may participate in acid-mediated regulation of YdiV expression. To test this, we constructed a *cadC* knockout strain of *Salmonella* (∆*cadC*) and then assessed the expression of *ydiV* using RT-PCR in WT and ∆*cadC* strains before and after acid treatment. The results showed that the expression of *ydiV* was markedly increased 2.49 times in the WT strain after acid treatment compared to the level before. However, in the ∆*cadC* strain after acid treatment, the expression of *ydiV* was slightly declined to 0.47 times the level before acid treatment (Figure 6). Overall, there was a 5.3-fold difference in expression of *ydiV* in the WT strain compared to the ∆*cadC* strain under the same acidic condition. This indicates that CadC plays an important role in *ydiV-*expression regulation under acidic condition since flagellum control during acid adaptation is dependent on YdiV and the expression of YdiV is induced by acid in a CadC-dependent manner. Next, flagellum growth was observed in WT, ∆*cadC*, and ∆*ydiV* strains under acidic conditions using negative-staining electron microscopy (Figure 7). Fewer flagella were observed in WT *Salmonella* cultured in pH 5.0; however, multiple flagellar filaments were observed in ∆*cadC and* ∆*ydiV* under pH 5.0 acidic condition (~6 flagella for ∆*cadC* and ~11 flagella ∆*ydiV*). The loss of the acid-related flagellum control phenotype in the ∆*cadC* and ∆*ydiV* strains indicated CadC and YdiV are key factors in the regulation of this process. Collectively, our results demonstrate that the CadC-YdiV axis is essential for acid-related flagellum control.

The transcription levels of YdiV in wild-type and ∆*ydiV* strains under normal or acid stress conditions were detected by qRT-PCR. Data are shown as means and SEM, n=3. ***, P < 0.001; **, P < 0.01; *, P < 0.05; n.s., P > 0.05.

(A to C) The numbers of flagella of the wild-type (A), ∆*cadC* (B) and ∆*ydiV* (C) strains were observed using negative-staining electron microscopy (EM). (D) Data are shown as the means and SEM, n=3. ***, P < 0.001; **, P < 0.01; *, P < 0.05; n.s., P > 0.05.

### CadC activates YdiV expression and flagellar control upon Salmonella entry into host cells.

**Figure 8.**
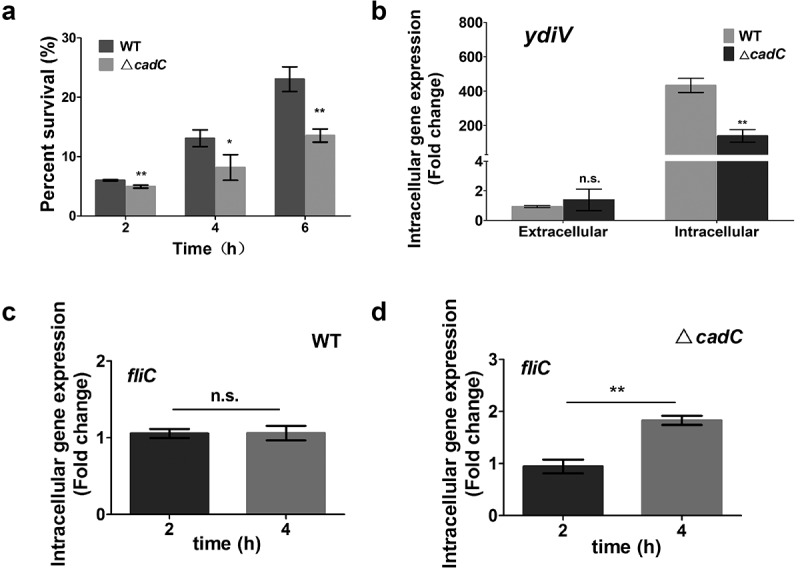
CadC is required for flagellar control of *Salmonella* within host cells.

Considering that CadC is a key regulator of *Salmonella* in response to acid stress, we speculate that it may affect *Salmonella* survival within host cells. Therefore, we used WT or ∆*cadC* to infect RAW264.7 cells with the same multiplicity of infection (MOI). At 2 h, 4 h and 6 h post-infection, RAW264.7 cells were lysed and then the number of bacteria in the cells was determined by counting CFU. The results showed that the survival rate of Δ*cadC* strain in RAW264.7 cells from 2 h to 6 h after infection were significantly lower than that of WT, suggesting CadC works in the whole infection period (Figure 8a). To further explore the mechanism of the effect of CadC on the intracellular survival of *Salmonella*, we used qRT-PCR to detect the transcription levels of *ydiV* and *fliC* in WT and Δ*cadC* strains at different post-infection time. Compared with that before invasion, the expression of *ydiV* in WT and Δ*cadC* strains after 4 h infection was significantly increased 433-fold and 139-fold, respectively. More importantly, the expression of *ydiV* in Δ*cadC* strain is only 1/3 of that in WT strain (Figure 8b). Then, the transcription level of *fliC* was detected 2 h or 4 h post infection. The results showed that the transcription level of *fliC* remained almost unchanged between 2 h and 4 h after WT *Salmonella* entering host cells, while the transcription of *fliC* increased significantly in Δ*cadC* strain at 4 h post infection than 2 h post infection (Figure 8c-d). To sum up, in host cells, CadC induces the expression of YdiV by sensing acid signals and then inhibits flagella synthesis, thus realizing *Salmonella* immune escape.

(A) Intracellular survival capability of WT and Δ*cadC* strains within RAW264.7 cells 2, 4, 6h post-infection. (B) The transcription levels of *ydiV* before and after *Salmonella* WT or ∆*cadC* invasion into RAW 264.7 cells were detected by qRT-PCR. (C) The mRNA levels of *fliC* were detected in WT by qRT-PCR at 2h and 4 h after WT *Salmonella* entry into RAW264.7 cells. (D) The mRNA levels of *fliC* were detected by qRT-PCR at 2h and 4 h after Δ*cadC* entry into RAW264.7 cells. Data are shown as means and SEM, n=3. ***, P < 0.001; **, P < 0.01; *, P < 0.05; n.s., P > 0.05.

## Discussion

Flagellum is an important motility organ, enabling movement of bacteria. The construction of bacterial flagella involves the synthesis of dozens of flagellar proteins in a highly energy-consuming process. In the initial stage of *Salmonella* infection, flagellum-mediated motility facilitates *Salmonella* gastrointestinal colonization of preferred sites.^48–51^ However, once *Salmonella* enters host cells, flagella are key in triggering of the host’s immune response. Interestingly, *Salmonella* can quickly turn off flagellum synthesis after entering host cells.^42,52^ How *Salmonella* senses the host cell environment and controls flagellum synthesis has not been fully determined.

After *Salmonella* entry, infected host cells recognize clues from the pathogens and undergo a series of changes, including acidification of the SCV.^53^ Therefore, acidic condition may be the signal that *Salmonella* perceives to sense the host environment. However, in different bacteria, there are varying effects of acid on flagellum synthesis. Previous research reported that *Escherichia coli* and *Vibrio parahaemolyticus* enhanced flagellum synthesis under acidic conditions.^54–56^ However, the regulation of flagellum synthesis in *Salmonella* under acidic conditions remains controversial. Some reports observed inhibited flagellum synthesis under acidic conditions, while another study has reported enhanced flagellum synthesis in *Salmonella* under acidic conditions.^57–60^ In this study, we determined that *Salmonella* effectively turns off flagellum synthesis under acidic conditions, which is consistent with the conclusions of earlier work. We further found that acid-related flagellum control occurred after *flhDC* transcription, and identified an important role of the anti-FlhDC factor YdiV. The acid-inducible transcription factor CadC can specifically bind to *ydiV* promoter as evidenced by DNA pull down of *Salmonella ydiV* promoter DNA with a whole-protein lysate of *Salmonella* cultured in acid condition. CadC is a previously characterized acid-inducible transcription factor that can regulate the expression levels of *cadA* and *cadB* genes. This is the first report of CadC-related regulation of the *ydiV* gene. Interestingly, a previous study reported that CadC increases the expression of FliC protein under acidic condition, which is opposite to our findings in this study.^59^ In that study, only weak movement of wild-type *Salmonella* was detected even at pH 7.0. Comparing the method of motility assay, we found that they used the overnight culture and then spotted on semisolid medium plates at different pH (10 mM lysine). While the wild-type *Salmonella* we used was in the logarithmic phase and the culture plate did not contain 10 mM lysine. The above reasons may lead to inconsistent experiment results.

**Figure 9.**
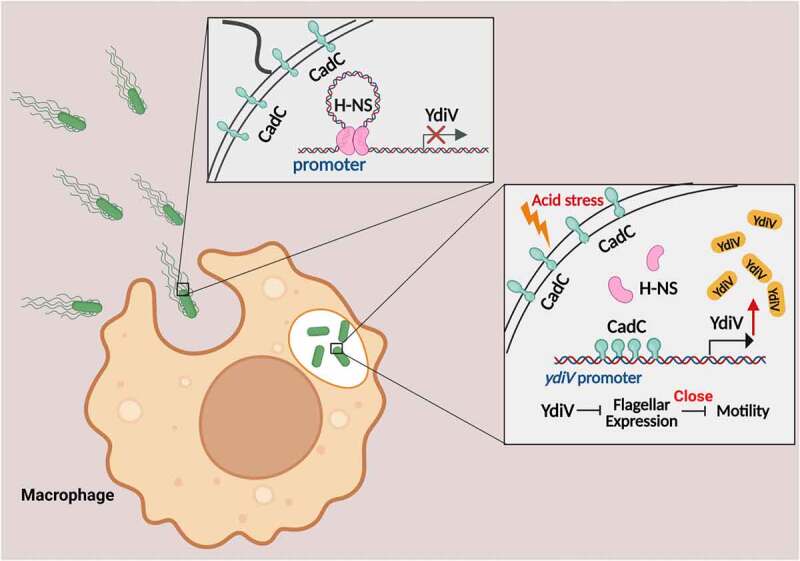
Model of acid-mediated flagellar control in *Salmonella.*

Our previous study showed that YdiV regulates flagellum biogenesis and iron absorption.^19,61^ Previous literature shows that H-NS mediates the silencing of *ydiV* gene under normal growth conditions.^62^ It has also been reported that YdiV expression is regulated by signals such as osmotic pressure and nutritional deficiency.^20,21^ This finding of the regulatory effect of acid on the expression of YdiV, and our results reveal CadC as an acid-sensing regulator of YdiV expression. Based on our experimental results, we propose a mechanistic model of acid-mediated flagellum control (Figure 9). Before *Salmonella* enters the host cell, H-NS binds to the promoter region of the *ydiv* gene to block transcription, so there is little expression of YdiV protein and *Salmonella* produce flagellum. Using their flagella, *Salmonella* can invade host cells. After *Salmonella* enters a host cell, the host cell acidifies rapidly. Under acidic conditions, CadC on the cell membrane is cut off by protease, releasing the DNA-binding domain of CadC into the cytoplasm. CadC then binds to the promoter region of *ydiV* with a high binding affinity, slowly replacing H-NS near the *ydiV* promoter, thus gradually relieving the expression inhibition of YdiV by H-NS. As a result, YdiV is expressed in large quantities. YdiV binds to FlhDC to shut-off the transcription of secondary and tertiary flagellar genes, and flagellum biogenesis is terminated. Although ∆*ydiV* has better mobility than WT under acidic conditions, it still has motility defect compared with mobility under pH 7.0. We speculate that it could be a second mechanism that also affects motility at acidic stress, which is independent of YdiV. In summary, our results indicate an acid-related flagellum control mechanism that is mainly mediated by CadC and YdiV. This regulation allows *Salmonella* to rapidly sense the host condition and turn off flagellar biogenesis to escape recognition by the immune system. *Salmonella* employs a complex mechanism to control flagellar synthesis. Under acidic conditions, the number of flagella in CadC knockout *Salmonella* was much greater than that of wild type *Salmonella*, but less than that of *ydiV* knockout *Salmonella*, suggesting that although CadC acts in the flagellar control of *Salmonella*, it might not be the only acid-mediated regulator. The results of our DNA pull-down experiment indicated that other transcription factors in addition to CadC may bind to the promotor of the *ydiV* gene to modulate expression of *ydiV*, suggesting that there is likely greater complexity of this regulatory network, which is worthy of further study.

Before *Salmonella* enters host cells, repressor H-NS inhibits the transcription of ydiV by binding to the promoter region to promote flagellar biogenesis. When *Salmonella* enters macrophage phagosomes and is subjected to acid stress signal, the membrane-bound transcriptional regulator CadC is activated by proteolytic cleavage. Binding of active CadC to the *ydiV* promoter releases the H-NS molecules, so CadC thereby functions as a transcriptional activator of *ydiV*. Increased levels of YdiV protein led to negative regulation of the flagellar master transcriptional activator complex, FlhD_4_C_2_, and subsequent downregulation of flagellar biogenesis.

## Material and methods

### Bacterial strains and growth conditions

*Salmonella typhimurium* ATCC 14028s was used for functional studies. Bacteria were routinely grown at 37°C in LB medium or Vogel and Bonner E medium with a final concentration of 0.4% glucose.^63^ When required, ampicillin was added to the culture medium to a final concentration of 100 μg/mL. For acid stress experiments, the bacteria were grown to OD_600_ = 0.4 in E glucose medium (pH 7.0), and then cultured under acidic condition (pH 5.0, 10 mM L-lysine). Unless otherwise specified, the acid used in acid stress experiments is acetic acid.

### Construction of CadC knock-out strain

The CadC knock-out strain was constructed with the one-step gene inactivation method as described previously.^64^

### Swimming motility

Swimming motility of bacteria strains was determined on semisolid E glucose medium containing 0.25% agar. Briefly, bacterial cells were cultured to OD_600_ = 0.4–0.6 and then adjusted to OD_600_ = 10 in PBS. The bacteria were inoculated on dry soft agar plates at different pH, after adjustment with acetic acid. The bacteria swimming diameters were measured after incubation at 30°C for 24 h.

### Negative-stain electron microscopy

To detect the effects of environmental pH changes on the number of flagella, the strains used in the motility assay were further analyzed by transmission electron microscopy. Cells were cultured at 37°C to early-logarithmic growth phase (OD_600_ = 0.4) in E glucose medium, followed by acid stress for 2 h. The cells were centrifuged at 5000 × *g* for 5 min at room temperature, and then suspended in 2.5% (w/v) glutaraldehyde. After dipping with carbon nets and then drying for 5 min in a natural state, samples were negatively stained with 1% phosphotungstic acid for 3 min. The samples were observed with a JEM-1200EX transmission electron microscope.

### RNA-sequencing analysis

The *Salmonella* wild-type strain was cultured overnight in LB medium. Cultures were diluted 1:100 in fresh E glucose medium (pH 7.0) and allowed to continue growth to OD_600_ = 0.4. Subsequently, half of the culture was treated with acid stress at pH 5.0 for 2 h and the remaining bacteria were further cultured. Next, 3 mL bacterial cells were obtained by centrifugation (3000 rpm, 5 min) and freezing treatment in liquid nitrogen. RNA isolation, library construction, and sequencing were performed by Novogene Co. Ltd. Total RNA samples were isolated from the obtained bacteria using TRIzol reagent according to the reagent manufacturer’s instructions. The quantification and qualification of total RNA was measured using 1% agarose gels and Agilent 2100 Bioanalyzer. The RNA was purified by removal of rRNA and random fragmentation before synthesis of cDNA. After adenylation of 3’ ends of DNA fragments, adaptors were ligated to prepare for hybridization. Finally, the library fragments were purified by AMPure XP system to preferentially select cDNA fragments 370–420 bp in length. PCR products were purified and library quality was assessed on the Agilent Bioanalyzer 2100 system. The libraries were sequenced on an Illumina Novaseq platform and 150 bp paired-end reads were generated.

### RNA preparation and real-time quantitative PCR

Total RNA was extracted with TRIzol reagent and reverse transcribed using the RevertAid cDNA Synthesis Kit according to the manufacturer’s instructions. Real-time quantitative PCR assay was performed using iTaq Universal SYBR Green SuperMix and corresponding primers with an Applied Biosystems 7500 Sequence Detection system. The relative mRNA expression of target genes was normalized to the expression level of GAPDH and the quantification result was calculated using the 2^−ΔΔct^ method.

### Western blot

Bacterial total proteins were heated at 95°C for 10 min and separated by SDS-PAGE. The proteins were transferred from gel onto a polyvinylidene fluoride (PVDF) membrane. After blocking with 5% milk in phosphate-buffered saline plus 0.1% Tween (PBST) at 25°C for 1 h, the membranes were incubated with primary antibodies in PBST overnight at 4°C. The membrane was then washed three times in PBST and incubated with secondary antibodies at 25°C for 1 h. After three washes with PBST, proteins on membranes were visualized with a chemiluminescent substrate and detected using a FluorChem imager. The protein levels were detected and quantified using ImageJ software. Primary antibodies against DnaK (Abcom, 1:10,000 dilution), FliC (Dia-An Biotech, 1:5,000 dilution), and YdiV (Dia-An Biotech, 1:2,000 dilution) were used. The secondary antibodies were anti-rabbit and anti-mouse IgG antibodies (Abcom, 1:10,000 dilution).

### Cell culture and bacterial infection experiments

The murine RAW264.7 macrophage-like cell line (ATCC) was cultured at 37°C with 5% CO_2_ in DMEM (Dulbecco's Modified Eagle Medium) medium supplemented with 10% fetal bovine serum (Gibco). The cells were seeded in triplicate in 100-mm tissue culture dishes at a cell density of 1 × 10^7^ cells per dish. *Salmonella* wild-type strain was cultured in LB medium at 37°C until reaching log-phase. The bacterial cells were then diluted in DMEM medium and seeded on RAW264.7 cells at a multiplicity of infection (MOI) of 10. After infection for 1 h, the cells were washed twice with PBS. Fresh DMEM medium with 100 µg/mL gentamicin was added to kill any remaining extracellular bacteria. At each time point post-infection, cells were washed and lysed in TRIzol reagent. The samples were stored at −70°C before RNA extraction. For the intracellular survival assays of bacteria strain within RAW264.7 cells, the cells were lysed with 1% Triton X-100, and the number of intracellular bacteria was detected by the colony-forming units (CFU) counts of viable colonies.

### Biotin-streptavidin pull-down

Proteins that bind the *ydiV* promoter were detected by biotin-streptavidin pull-down assay as previously described.^65–67^ Briefly, the DNA sequence of *ydiV* promoter was amplified by PCR and labeled with biotin using Biotin DecaLabel DNA Labeling Kit. Next, 5 ug biotin-labeled *ydiV* promoter DNA probes and 500 ug nuclear protein extracts from bacteria cells were mixed and incubated on ice. After washing with PBS buffer, 100 µL Streptavidin-agarose G beads were added into the DNA-nuclear protein mixture and incubated for 1 h at 4°C. Beads were then collected by centrifugation and washed three times with PBS buffer. The protein-DNA-streptavidin-agarose complexes were identified by Western blotting and mass spectrometry.

The “bacterial extract” used in the above experiment is prepared as follows. *Salmonella typhimurium* ATCC 14028s were grown at 37°C in E glucose medium (pH 7.0) to OD_600_ = 0.4. Subsequently, half of the culture was treated with acid stress at pH 5.0 for 2 h and the remaining bacteria were further cultured. Cells were then resuspended in PBS buffer and disrupted by high pressure systems at 4°C. After centrifugation at 14,000 rpm for 60 min to remove debris, the supernatant used as the bacterial extract.

### Electrophoretic mobility shift assay

EMSA was performed by the method described.^19^ Briefly, 5 µM DNA was incubated with different ratios of proteins in reaction buffer (20 mM Tris–HCl, 100 mM NaCl, 1 mM MgCl_2_, 1 mM ZnCl_2_ and 4%(v/v) glycerol, pH 8.0) for 10 min at room temperature. Samples were separated on native 5% polyacrylamide gel and then stained using GelRed and Coomassie brilliant blue.

For EMSA competition assay, 5 µM *ydiV* promoter DNA was incubated with 30 µM H-NS for 30 min at 4°C. Then, increasing amounts of the CadC_c_ protein (3.75 to 150 μM) was added. After incubation at 4°C for 30 minutes, samples were separated on native 5% polyacrylamide gel and then stained using GelRed and Coomassie brilliant blue.

### DNase I footprinting assay

DNase I footprinting assays were performed similarly to the method described by Wang et al.^68^ For preparation of fluorescent FAM labeled probes, the *ydiV* promoter region was PCR amplified using primers of *ydiV* promoter-5 (FAM) and ydiV promoter-3. After purification and quantification, 300 ng FAM-labeled probes were incubated with different amounts of proteins (CadCc or H-NS) for 30 min at 30°C in a total volume of 40 µL. The protein-probe mixture was then added 10 µL reaction buffer, which contained about 0.015 unit DNase I and 100 nmoL freshly prepared CaCl_2_ solution, and then incubated at 37°C for 1 min. The reaction was stopped by the addition of 140 µL DNase I stop buffer (200 mM unbuffered sodium acetate, 30 mM EDTA, and 0.15% SDS). Samples were extracted with phenol/chloroform and then precipitated with ethanol. The obtained pellets were dissolved in 30 µL Milli-Q water and used for DNA ladder preparation and electrophoresis. The obtained data were analyzed using Peak Scanner software v1.0.

### Biolayer interferometry assay

Biolayer interferometry (BLI) assay was performed using an Octet RED96 instrument at 25°C.^69^ To examine the interaction between CadCc and the overlapping DNA fragment, the DNA was biotin-conjugated at the C-terminus and diluted to 1 μM in buffer A (PBS buffer containing 1 mM ZnCl_2_). Following an initial baseline step in buffer A, the streptavidin sensor captured biotin-conjugated DNA at a ligand density of ~1 nm. Following equilibration in buffer A for 60 s, the ligand-coated sensor was transferred to buffer B (buffer A containing 50 µg/mL CadCc) for 180 s followed by buffer A for 180 s to determine the binding/association signal. The unloaded DNA fragment sensor was used as the control group. The interaction between H-NS and the overlapping DNA fragment was detected by the same method, and the buffer A used in the process was PBS buffer.

### Plasmid construction

DNA fragments of CadCc and H-NS genes were amplified by PCR from genomic DNA from *E. coli* strain K-12 substrain MG1655. After digestion with restriction endonuclease BamH I and Xho I, the PCR fragments were inserted into the pGL01 vector, the resulting recombinant plasmids pGL01-CadCc or pGL01-H-NS, which has 6 × His-tag at the N terminal of the protein. CadCc mutants (R7A, R32A, H42A, H43A, R60A, and W106A) were constructed using a site-directed mutagenesis system. Briefly, the DNA fragments of CadCc mutants gene were amplifified by PCR from recombinant plasmid pGL01-CadCc. After digestion with Dpn I, the linear PCR fragments were ligated and cyclized to generate plasmids pGL01-CadC_mut_. All plasmids and primers used in this study are listed in Table S2 and S3.

### Protein expression and purification

*E. coli* BL21 (DE3) was used to express the CadCc and H-NS proteins from the corresponding plasmids. *E. coli* cells were grown in LB media containing 100 μg/mL ampicillin at 37°C and protein expression was induced overnight by addition of 0.6 mmol/L isopropyl β-D-1- thiogalactopyranoside (IPTG) at 16°C. Cells were then harvested and disrupted. Proteins were purified by Ni^2+^ His-Trap HP, and determined by SDS-PAGE followed by coomassie blue staining. All protein purification steps were performed at 4°C.

### CadCc–DNA molecular modeling

Docking simulations between CadCc and DNA were performed and measured using Mode Tec, Inc. (Sichuan, China) based on the CadC structure and molecular docking. The structure of the *E.coli* CadC DNA binding domain (PDB code: 5JU7) was used to model the *Salmonella* CadC structure by homology modeling.^70^

## Supplementary Material

Supplemental Material

## Data Availability

The data that support the findings of this study are available from Bingqing Li (bingqingsdu@163.com), upon reasonable request. Original data is available in Mendeley Dataset (http://doi.org/ 10.17632/r733bshdnv.1) and GEO (https://www.ncbi.nlm.nih.gov/geo/query/acc.cgi?acc=GSE210570).
