# Supplementary material for: Host acid signal controls Salmonella flagella biogenesis through CadC-YdiV axis
Source: Gut Microbes. 2022 Dec 1;14(1):2146979. doi: 10.1080/19490976.2022.2146979 (PMC9728131; doi:10.1080/19490976.2022.2146979)
Supplement: Supplemental Material [file KGMI_A_2146979_SM1625.pdf]

SUPPLEMENTAL MATERIALS

Host Acid Signal Controls *Salmonella* Flagella Biogenesis through CadC-YdiV Axis

Weiwei Wang[1,2], Yingying Yue[1,2], Min Zhang[2], Nannan Song[1,2], Haihong Jia[1,2], Yuanji Dai[2], Fengyu Zhang[3], Cuiling Li[1,2] and Bingqing Li[1,2,4,5]*

[1] Department of Clinical Laboratory, Shandong Provincial Hospital Affiliated to Shandong First Medical University, Jinan, 250021, Shandong, China;

[2] Department of Pathogen Biology, School of Clinical and Basic Medical Sciences, Shandong First Medical University & Shandong Academy of Medical Sciences, Jinan, 250062, China.

[3] State Key Laboratory of Microbial Technology, School of Life Sciences, Shandong University, Qingdao, 266237, China.

[4] Key Lab for Biotech-Drugs of National Health Commission, Jinan 250062, Shandong, China;

[5] Key Lab for Rare & Uncommon Diseases of Shandong Province, Jinan, 250062, Shandong, China.

*Correspondence: bingqingsdu@163.com

Figure S1-S8

Table S1-S3

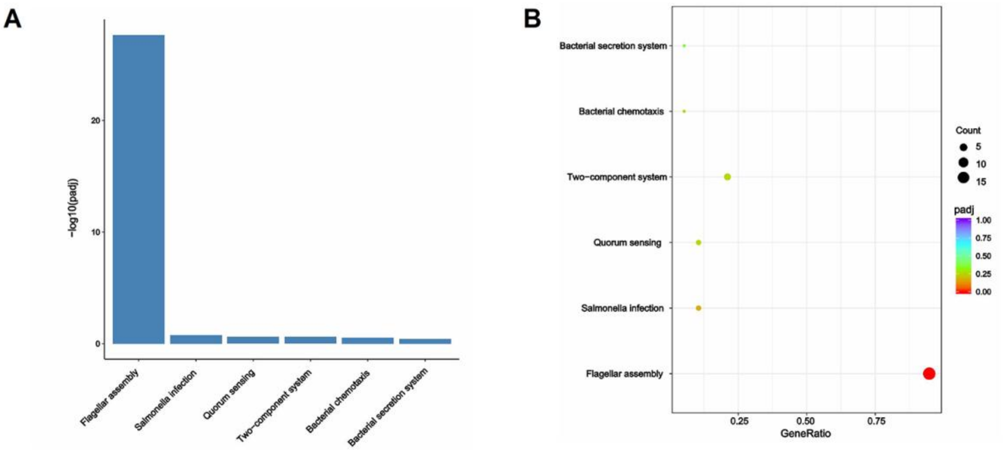

FIG S1. KEGG enrichment analysis of the *Salmonella* pathways regulated under the acid stress condition.

Related biological function of all 6 pathways are displayed in bar (A) and dot (B), and bar height or dot size indicates the level of significance of enrichment.

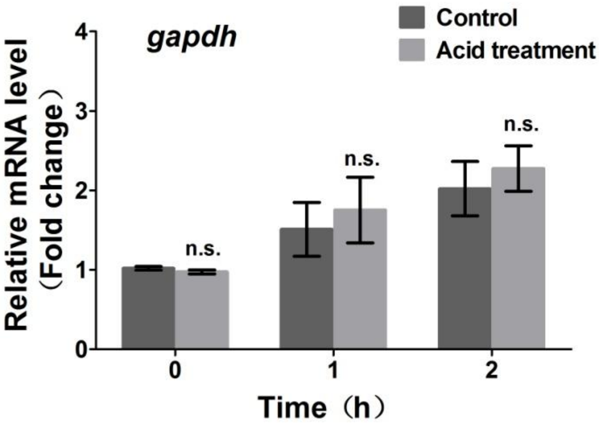

FIG S2. The relative transcription levels of *gapdh* under acid stress condition.

The mRNA levels of *gapdh* in wild-type strain cultured in pH 5.0 condition, compared with the one in pH 7.0. The *gmk* (guanylate monophosphate kinase) gene was used as an internal control.

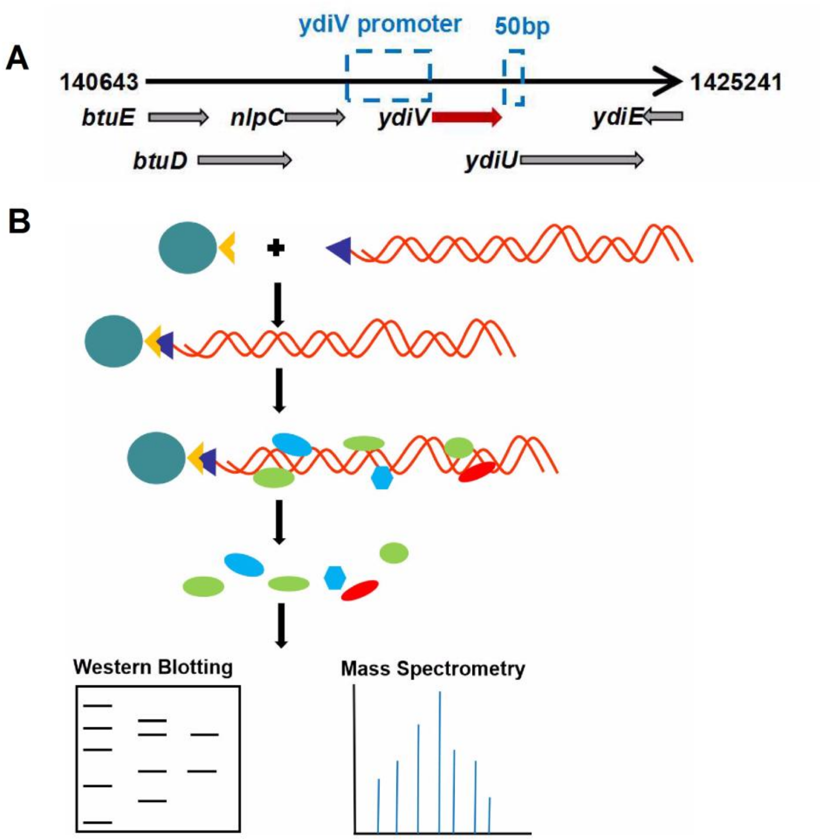

FIG S3. Schematic of the *ydiV* promoter genomic position and streptavidin pull-down approach.

(A) The *ydiV* promoter is a base sequence located in the genome between the *ydiV* and *nlpC* genes, this region is marked with a blue box. (B) A specific DNA probe was designed for the *ydiV* promoter region and labeled with desthiobiotin, which could bind to streptavidin coupled to magnetic beads. Then, the cell extract is incubated with the magnetic bead-DNA probe, and the target protein molecule can specifically bind to the DNA probe. Finally, the protein is identified by Western Blot or mass spectrometry (MS).

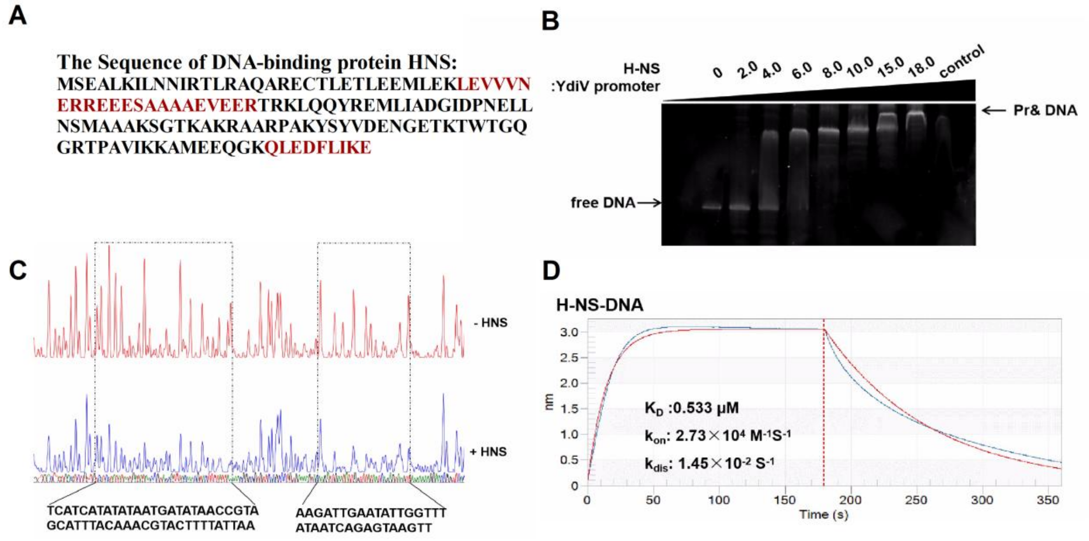

FIG S4. H-NS binds to the promoter region of *ydiV*.

(A) Amino acid sequence of H-NS. Red sequence indicates the fragment identified by MS. (B) EMSA result for H-NS and *ydiV* promoter DNA; 5 μM *ydiV* promoter DNA was pre-incubated with different ratios of H-NS for 10 min. Mixtures were analyzed by native 5% polyacrylamide gel at 4 °C and then stained with GelRed. The ratio of protein to DNA ranged from 0 to 18. A sample containing only H-NS was used as a control. (C) DNase I footprinting assays of the *ydiV* promoter with H-NS. The promoter fragment of *ydiV* was PCR amplified and labeled with FAM, incubated with increasing amounts of purified H-NS, and then subjected to DNase I footprinting assay. DNase I digestion reactions were analyzed by ABI 3500XL DNA analyzer. Protected regions are boxed and marked with positions. The upper picture shows the control reactions (no added protein). Protected regions are shown in dotted squares. (D) Biolayer interferometry (BLI) analysis of the binding capacity of H-NS to overlapping DNA. Assessment of BLI response signal (nm) for H-NS protein binding to DNA.

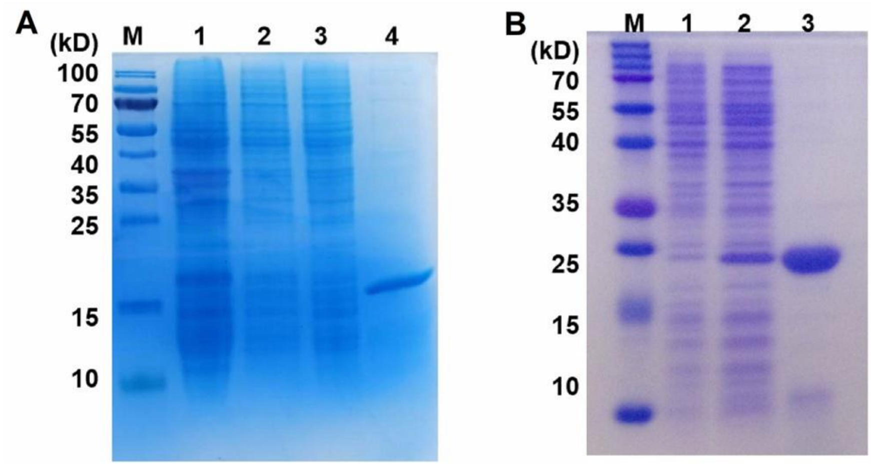

FIG S5. The expression and purification of CadC$_C$ and H-NS proteins.

Protein samples were separated by SDS-PAGE and protein levels are shown after coomassie blue staining. (A) H-NS protein. Lane M, protein marker; lane 1, the sample from the supernatant of cell lysate after induction; lane 2 and 3, flowthrough sample after Ni-NTA affinity column; lane 4, purified H-NS. (B) CadC$_c$ protein. Lane M, protein marker; lane 1, flowthrough sample after Ni-NTA affinity column; lane 2, the sample from the supernatant of cell lysate after induction; lane 3, purified CadC$_c$. These two proteins were from *E. coli*.

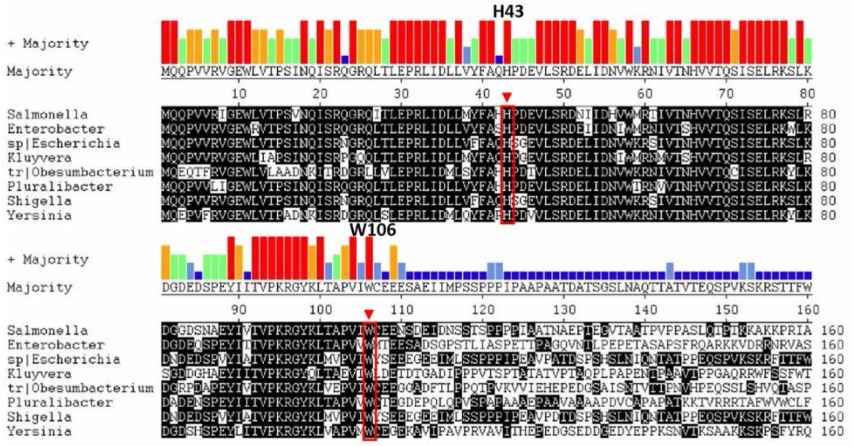

FIG S6. Sequence alignment of CadC$_C$ from 8 different bacteria.

One hundred and sixty residues (1–160 aa) of CadC are used in this alignment, the key residues H43 and W106 for the interaction between CadC$_c$ and *ydiV* promoter are highly conserved. The key residues H43 and W106 highlighted by red box.

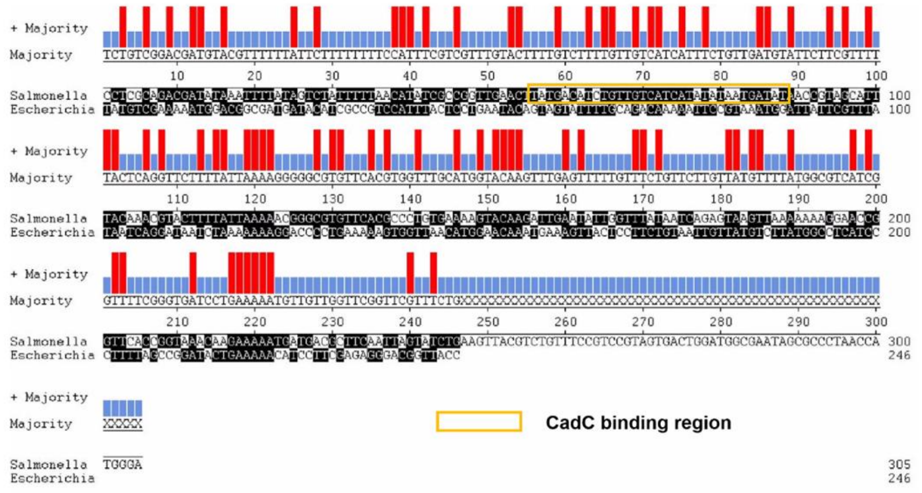

FIG S7. Sequence alignment of *ydiV* promoter between *Salmonella* and *E. coli*. The $CadC_c$ binding region of the *Salmonella ydiV* promoter, which highlighted by yellow box, showed not significant sequence conservation with *E. coli*.

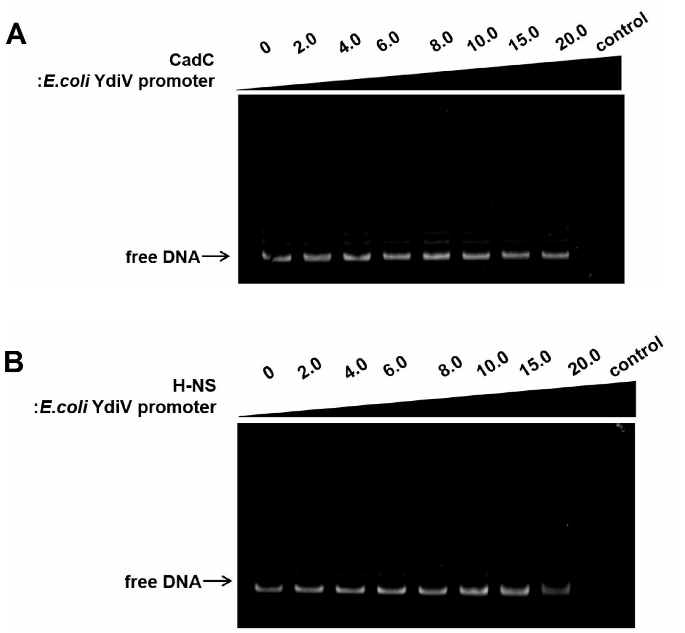

FIG S8. EMSA results of $CadC_C$ and H-NS with *E.coli ydiV* promoter DNA.

5 μM *E.coli ydiV* promoter DNA was pre-incubated with different ratios of protein for 10 min. Mixtures were analyzed by native 5% polyacrylamide gel at 4 °C and then stained with GelRed. The ratio of protein to DNA ranged from 0 to 20. A sample containing only $CadC_C$ or H-NS was used as a control. (A) EMSA of $CadC_C$ and *E.coli ydiV* promoter DNA. (B) EMSA of H-NS and *E.coli ydiV* promoter DNA.

Table S1. Strains used in this study

No	Strains	Relevant characteristic(s)	Source
1	WT *Salmonella*	*Salmonella enterica serovar Typhimurium* ATCC14028, no resistance	American Type Culture Collection
2	*Salmonella* Δ*cadC*	*cadC* knockout strain, no resistance	This study
3	*Salmonella* Δ*ydiV*	*ydiV* knockout strain, no resistance	[1]
4	*E. coli* BL21(DE3)	T7 expression host, no resistance	Takara Bio Inc.
5	*E. coli* BL21(DE3)p*cadC*$_c$	CadC$_c$/pGl01, Amp$^+$	This study
6	*E. coli* BL21(DE3)p*hns*	H-NS/pGl01, Amp$^+$	This study
7	*E. coli* BL21(DE3)p*cadCR7A*	CadCR7A/ pGl01, Amp$^+$	This study
8	*E. coli* BL21(DE3)p*cadCR32A*	CadCR32A/ pGl01, Amp$^+$	This study
9	*E. coli* BL21(DE3)p*cadCH42A*	CadCH42A/ pGl01, Amp$^+$	This study
10	*E. coli* BL21(DE3)p*cadCH43A*	CadCH43A/ pGl01, Amp$^+$	This study
11	*E. coli* BL21(DE3)p*cadCR60A*	CadCR60A/ pGl01, Amp$^+$	This study
12	*E. coli* BL21(DE3)p*cadCW106A*	CadCW106A/ pGl01, Amp$^+$	This study

[1] Y. Yue, W. Wang, Y. Ma, N. Song, H. Jia, C. Li, Q. Wang, H. Li, and B. Li, under review.
The *ydiV* knockout strain was constructed using the lambda Red recombinase system. The resistance cassette was moved.

Table S2. Plasmids used in this study

No	Plasmids	Relevant characteristic(s)	Source
1	pGL01	Expression Vector Amp$^+$	[3]
2	CadC$_c$/pGL01	CadC 1-160aa cloned into pGL01	This study
3	H-NS/pGL01	H-NS cloned into pGL01	This study
4	CadC R7A/pGL01	CadC$_c$ R7A mutant protein expression construct into pGL01 vector	This study
5	CadC R32A/pGL01	CadC$_c$ R32A mutant protein expression construct into pGL01 vector	This study
6	CadC H42A/pGL01	CadC$_c$ H42A mutant protein expression construct into pGL01 vector	This study
7	CadC H43A/pGL01	CadC$_c$ H43A mutant protein expression construct into pGL01 vector	This study
8	CadC R60A/pGL01	CadC$_c$ R60A mutant protein expression construct into pGL01 vector	This study
9	CadC W106A/pGL01	CadC$_c$ W106A mutant protein expression construct into pGL01 vector	This study

[1] Li, B., Li, N., Wang, F., Guo, L., Huang, Y., Liu, X., ... & Gu, L. (2012). Structural insight of a concentration-dependent mechanism by which YdiV inhibits *Escherichia*

coli flagellum biogenesis and motility. *Nucleic acids research*, 40(21), 11073-11085.

Table S3. Primers used in this study

No	Oligonucleotide	Sequence	Application
1	CadC$_c$-pGl01-F	ataggatccatgcagcaacctgttgtacgc	Gene Clone
2	CadC$_c$-pGl01-R	atactcgagttaagcggcaatacgcggctt	Gene Clone
3	H-NS-pGl01-F	ataggatccatgagcgaagcacttaaaatt	Gene Clone
4	H-NS-pGl01-R	atactcgagttattccttgatcaggaaatcttc	Gene Clone
5	CadC-R7A-F	tgttgtaGCCattggagagtggctggttacacc	Gene Clone
6	CadC-R7A-R	ctccaatGGCtacaacaggttgctgcatggat	Gene Clone
7	CadC-R32A-F	ttgaaccaGCCctgatcgatcttctgatgtattttgc	Gene Clone
8	CadC-R32A-R	gatcagGGCtggttcaagagtaatctggcgtc	Gene Clone
9	CadC-H42A-F	tgtattttgcgGCCcacccggatgaagtgttaagcc	Gene Clone
10	CadC-H42A-R	gtgGGCcgcaaaatacatcagaagatcgatca	Gene Clone
11	CadC-H43A-F	tattttgcgcatGCCccggatgaagtgttaagccg	Gene Clone
12	CadC-H43A-R	ggGGCatgcgcaaaatacatcagaagatcgat	Gene Clone
13	CadC-R60A-F	tcatgtctggatgGCCaccatcgtgaccaaccatgttg	Gene Clone
14	CadC-R60A-R	tGGCcatccagacatgatcaataatgttatcc	Gene Clone
15	CadC-W106A-F	gttatcGCCtgcgaagaaaatagcgatgagat	Gene Clone
16	CadC-W106A-R	tcttcgcaGGCgataaccggcgccgtcagctt	Gene Clone
17	*flhD*-5	cgcctcggtatcaacgaaga	qPCR
18	*flhD*-3	ctccgccagtttgaccatct	qPCR
19	*fliA*-5	cttacccagtttggtgcg	qPCR
20	*fliA*-3	cgagcaactggtgttaac	qPCR
21	*fliC*-5	cgcagtaaagagaggacg	qPCR
22	*fliC*-3	gggcaacaccgtaaacaacc	qPCR
23	*gap*-5	gaccttcgatgatgccgaag	qPCR
24	*gap*-3	gccaggacatcgtttccaac	qPCR
25	*ydiV*-5	cagcagcgagctgaaatgatc	qPCR
26	*ydiV*-3	cgcaaacatcgcctcagtatc	qPCR
27	*ydiV* promoter-5	cctcgcagacgatataaattttatagtc	EMSA and Biotin-streptavidin pull-down Assay
28	*ydiV* promoter-3	tacgccagtcccatggttagggcgc	EMSA and Biotin-streptavidin pull-down Assay
29	overlap DNA-5	tcatcatatataatgatat	EMSA
30	overlap DNA-3	atatcattatatatgatga	EMSA
31	*ydiV* promoter-5	190(FAM)-cctcgcagacgatataaatt	DNase I footprinting assay
32	*ydiV* promoter-3	190-tacgccagtcccatggttag	DNase I footprinting assay
33	overlap DNA-5	biotin-tcatcatatataatgatat	Biolayer interferometry assay
34	overlap DNA-3	atatcattatatatgatga	Biolayer interferometry assay
